# Targeting BMK1 Impairs the Drug Resistance to Combined Inhibition of BRAF and MEK1/2 in Melanoma

**DOI:** 10.1038/srep46244

**Published:** 2017-04-07

**Authors:** Chengli Song, Lina Wang, Qiang Xu, Kai Wang, Dan Xie, Zhe Yu, Kui Jiang, Lujian Liao, John R. Yates, Jiing-Dwan Lee, Qingkai Yang

**Affiliations:** 1 Department of Oncology, Second Affiliated Hospital, Institute of Cancer Stem Cell, DaLian Medical University, 9 Western Lvshun South Road, Dalian, Liaoning 116044, China; 2 Shanghai Key Laboratory of Regulatory Biology, School of Life Sciences, East China Normal University, Shanghai, 200241, China; 3 Department of Chemical Physiology, The Scripps Research Institute, 10550 North Torrey Pines Road, La Jolla, CA 92037, USA; 4 Department of Immunology and Microbial Science, The Scripps Research Institute, 10550 North Torrey Pines Road, La Jolla, CA 92037, USA

## Abstract

Combined inhibition of BRAF and MEK1/2 (CIBM) improves therapeutic efficacy of BRAF-mutant melanoma. However, drug resistance to CIBM is inevitable and the drug resistance mechanisms still remain to be elucidated. Here, we show that BMK1 pathway contributes to the drug resistance to CIBM. Considering that ERK1/2 pathway regulates cellular processes by phosphorylating, we first performed a SILAC phosphoproteomic profiling of CIBM. Phosphorylation of 239 proteins was identified to be downregulated, while phosphorylation of 47 proteins was upregulated. Following siRNA screening of 47 upregulated proteins indicated that the knockdown of BMK1 showed the most significant ability to inhibit the proliferation of CIBM resistant cells. It was found that phosphorylation of BMK1 was enhanced in resistant cells, which suggested an association of BMK1 with drug resistance. Further study indicated that phospho-activation of BMK1 by MEK5D enhanced the resistance to CIBM. Conversely, inhibition of BMK1 by shRNAi or BMK1 inhibitor (XMD8-92) impaired not only the acquirement of resistance to CIBM, but also the proliferation of CIBM resistant cells. Further kinome-scale siRNA screening demonstrated that SRC\MEK5 cascade promotes the phospho-activation of BMK1 in response to CIBM. Our study not only provides a global phosphoproteomic view of CIBM in melanoma, but also demonstrates that inhibition of BMK1 has therapeutic potential for the treatment of melanoma.

Activation of ERK1/2 (extracellular signal-regulated kinase 1 and extracellular signal-regulated kinase 2) pathway by mutant BRAF (B-Raf proto-oncogene, serine/threonine kinase) is found in above 50% patients with advanced melanoma^1^,^2^. Inhibition of BRAF with the small molecule inhibitor such as Vemurafenib (PLX4032), has shown impressive initial responses in patients with BRAF mutant melanoma^3^. Unfortunately, acquired resistance to anti-BRAF monotherapy frequently develops from several drug resistance mechanisms, which mainly reactivate the ERK1/2 pathway^4^,^5^,^6^. Based on these resistance mechanisms, combination of BRAF and MEK1/2 inhibitors (such as Vemurafenib plus Trametinib) is applied for BRAF mutant melanoma. The combined inhibition of BRAF and MEK1/2 (CIBM) indeed bypasses some resistance mechanisms and results in a improvement^7^,^8^. However, the failure due to drug resistance is still inevitable^7^,^8^, which reinforces the importance of understanding the drug resistant mechanisms. Since CIBM should significantly reduce possibility of the reactivation of ERK1/2 pathway, one reason for CIBM drug resistance might be the induction of counteracting pathways in responses to CIBM^8^,^9^,^10^.

Although ERK1/2 pathway regulates cellular processes through phosphorylating^11^, most studies about the drug resistance to the inhibition of ERK1/2 pathway were focused on the gene expression^9^,^10^. In the meantime, the counteracting phospho-activation of multi survival pathways has been supposed to be the most critical resistance mechanisms in melanoma^8^,^9^,^10^. Despite recent progresses of the resistance mechanisms in melanoma, little is known about phosphoproteomic profiling of the inhibition of ERK1/2 pathway. SILAC (stable isotope labeling with amino acids in cell culture) provides a strategy to label the proteins with different stable isotopic forms of the amino acids. Cells are labeled during the culturing process using media containing light or heavy amino acids, the heavy amino acids have stable isotope atoms incorporated e.g. [U-^13^C_6_]-L-lysine and [U-^13^C_6_, ^15^N_4_]-L-arginine. The labeled amino acids are used in protein synthesis, after several passages the labeled residues have been fully incorporated into the proteins. The labeled amino acids are equivalent to their unlabeled counterparts. Consequently, it is possible to monitor quantitative phospho-differences at the protein level between control and CIBM treatment cells.

In addition, four MAP kinase pathways have been identified in mammal: ERK1/2, BMK1 (mitogen-activated protein kinase 7 or big mitogen-activated protein kinase 1), p38 (mitogen-activated protein kinase 14) and JNK (mitogen-activated protein kinase 8) pathways (Fig. 1a)^11^. Activated by growth factors, ERK1/2 and BMK1 pathways promote cell proliferation and survival. While JNK and p38 pathways regulate cell death and proliferation^11^,^12^. Among the MAPKs, BMK1 is the most similar to ERK1/2. Hence, it is not surprising that BMK1 share a bunch of substrates with ERK1/2^11^,^12^. In response to extracellular signals, BMK1 is specifically activated by MEK5 (mitogen-activated protein kinase kinase 5) and translocates to the cell nucleus and regulates gene expression by phosphorylating^12^,^13^. Like ERK1/2 pathway, BMK1 pathway has been reported to play critical roles in the multi properties of human malignancies, including tumorigenesis, chemoresistance^14^, proliferation^15^ and metastasis^16^. In the previous study^13^, a small molecular inhibitor of BMK1, XMD8-92, was developed. This BMK1 inhibitor, XMD8-92, can efficiently suppress the proliferation of multi types of cancer cells^13^,^17^.

In this study, we not only provide a global phosphoproteomic profiling of CIBM, but also demonstrate that targeting BMK1 impairs the drug resistance and BMK1 inhibitor–XMD8-92 might have therapeutic potential for the treatment of melanoma.

## Results

### Phosphoproteomic profiling revealed that the upregulated phosphosites were enriched in tyrosine sites and MAPK pathways

Since ERK1/2 pathway is well known to control cellular processes through phosphorylating, SILAC phosphoproteomic profiling was carried out to identify the counteracting pathways regulated by phosphorylation. Briefly, A375 melanoma cells grown in light medium without serum were treated with Vemurafenib (BRAF inhibitor) and Trametinib (MEK1/2 inhibitor), while cells grown in heavy medium without serum were treated with vehicle. Then these two populations of cells were treated with serum for 1.5 hrs. The 1.5-hour treatment was used to amplify the counteracting phosphorylation in this study, while 10–20 minter treatment is generally used to detect direct phosphorylation in most studies. The resultant proteins from two populations were mixed at 1:1 ratio and analyzed by mass spectrometer.

**Figure 1:**
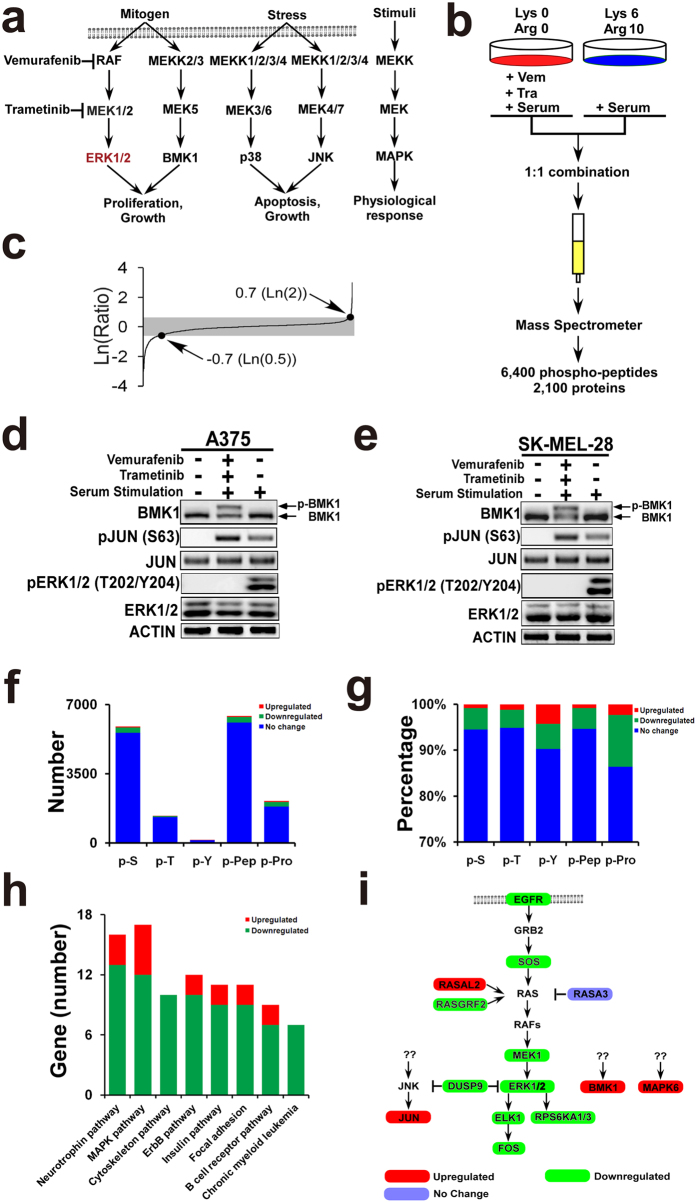
Phosphoproteomic profiling of CIBM. (**a**) Mitogen-activated protein kinase (MAPK) signalling cascades in mammalian cells. (**b**) Experimental scheme for phosphoproteomic profiling. Light cells were pretreated with 2 μM Vemurafenib and 10 nM Trametinib for 1 h. After treated with serum for 1.5 hrs, heavy and light cell lysates were combined in a 1:1 ratio. This was followed by mass spectrometer analysis, which resulted in about 6,400 phosphosites identified. (**c**) Distribution of the ratio of phosphopeptides detected in phosphoproteomic profiling. (**d**,**e**) A375 and SK-MEL-28 cells were serum starved overnight followed by treatment with/without 2 μM Vemurafenib and 10 nM Trametinib for 1 hr as noted. Then cells were stimulated with serum for 1.5 hrs and phosphorylated BMK1 was detected by mobility retardation^27^. ACTIN, JUN, phospho-JUN, ERK1/2 and phospho-ERK1/2 (T202/Y204) were detected by the antibody as noted. (**f**) Number of phosphosites (p-S, p-T and p-Y), phosphopeptides (p-Pep), and phosphoproteins (p-Pro) identified in this study. p-S: phosphorylated serine; p-T: phosphorylated threonine; p-Y: phosphorylated tyrosine. (**g**) Percentage of phosphosites (p-S, p-T and p-Y), phosphopeptides (p-Pep), and phosphoproteins (p-Pro) identified. p-S: phosphorylated serine; p-T: phosphorylated threonine; p-Y: phosphorylated tyrosine. (**h**) Number of gene enriched according to pathways by DAVID Bioinformatics Resources 6.7 using default setting. (**i**) Network scheme of MAPK pathways shows the emergent signaling, which occurs when BRAF-MEK is inhibited.

Following the previous study^18^, we identified 6432 phosphosites derived from 2123 proteins (Fig. 1b) (Supplementary Table S1). 342 phosphosites with over two fold change were considered significant and used for the further analysis (Fig. 1c). Western blotting was applied to validate the phospho-change of these phosphosites. Consistent with the results of mass spectrometry, western blotting data indicated that the phosphorylation of ERK1/2 was inhibited, while the phosphorylation of BMK1 and JUN was upregulated (Fig. 1d,e). Furthermore, phosphorylation of 289 sites from 239 proteins was identified to be significantly downregulated (Supplementary Table S2), while phosphorylation of 53 sites from 47 proteins was upregulated (Supplementary Table S3) (Fig. 1f). In other words, phosphorylation of 4.2% phosphosites was downregulated, while phosphorylation of 0.8% phosphosites was upregulated (Fig. 1g).

To investigate the potential roles of these phospho-changes, we performed enrichment analysis of phosphosite (Fig. 1g), pathway (Fig. 1h), subcellular localization (Supplementary Fig. S1a and S1b), molecular function (Supplementary Fig. S1c and S1d), and biological process (Supplementary Fig. S1e and S1f). Chi-square test was used to evaluate whether the enrichment is more than random chance compared with the background (6432 phosphosites or 2123 proteins). It was found that the upregulated phosphosites was significantly enriched in p-Y (phospho-tyrosine) (*p* value 0.0049). The enrichment of p-Y is side supported by the previous studies— some tyrosine kinases (such as receptor tyrosine kinases) promote the drug resistance^19^,^20^,^21^. As expected, the phosphorylation of numerous kinases was remarkable decreased by CIBM (*p* value 0.0097). Interestingly, enrichment analysis indicated that not only downregulated phospho-proteins (*p* value 0.0079) but also upregulated phospho-proteins (*p* value 0.0045) showed significant enrichment in MAPK pathways (Fig. 1i). It was found that the phosphorylation of ERK1/2 (Thr202/Tyr204) was inhibited as expected, while the phosphorylation of BMK1 and JUN was upregulated. Because the phospho-activation of multi pathways is taken as one of the most important resistance mechanisms^8^,^9^,^10^, phospho-activation of parallel MAPK pathways might be critical in drug resistance.

### siRNAi of upregulated proteins indicated that knockdown of BMK1 impairs the proliferation of CIBM drug resistant cells

**Figure 2:**
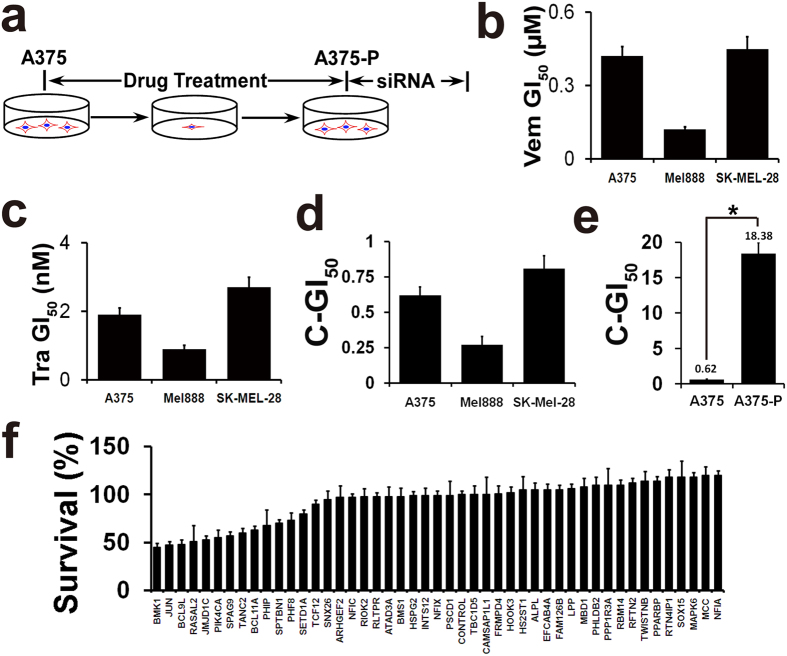
Knockdown of BMK1 impairs the proliferation of drug resistant cells. (**a**) Experimental scheme for A375-P drug resistant cells. Briefly, A375 cells were treated with 2 μM Vemurafenib and 10 nM Trametinib. The combined GI_50_ of A375 and A375–P cells were used to confirm the resistance to Vemurafenib and Trametinib. (**b**) The GI_50_ (concentration which inhibits growth by 50%) of Vemurafenib was evaluated in A375, Mel888 and SK-MEL-28 cells. Data from growth-inhibition assays were modeled using a nonlinear regression curve fit with a sigmoid dose–response. In this study, n = 6, ±SEM and the GI_50_ generated by GraphPad Prism5 were applied unless otherwise stated. (**c**) The GI_50_ of Trametinib in A375, Mel888 and SK-MEL-28 cells. (**d**) The combined GI_50_ of Vemurafenib and Trametinib in A375, Mel888 and SK-MEL-28 cells. The combined GI_50_ was assessed by treating cells with series fold BCVT. For example, two fold BCVT is 0.8 μM Vemurafenib and 4 nM Trametinib. Similarly, ten fold BCVT is 4 μM Vemurafenib and 20 nM Trametinib. (**e**) The combined GI_50_ of Vemurafenib and Trametinib in A375 and A375–P cells. Unless otherwise stated, *p < 0.05 was taken as significant. (**f**) Effect of upregulated p phospho-rotein knock down on the proliferation of A375-P drug resistant cells. Five replicates of A375-P cells were transfected with siRNA or control siRNAs for 72 hrs. Then cell number was evaluated by MTT assays.

Since the counteracting phospho-activation of survival pathways is critical in drug resistance^8^,^9^,^10^, we hypothesized that the upregulated phospho-proteins potentially promote the drug resistance. To validate the role of these upregulated proteins, siRNA screening was used to evaluate their roles in the proliferation of A375-P (post Vemurafenib and Trametinib treatment) drug resistant cells (Fig. 2a). To normalize the resistance effects to CIBM, the GI_50_ (concentration which inhibits growth by 50%) of Vemurafenib and Trametinib was first assessed. As shown in Fig. 2b,c, the A375 GI_50_ of Vemurafenib and Trametinib were about 0.4 μM and 2 nM, respectively. To facilitate the assay of combined GI_50_ (C-GI_50_) of Vemurafenib and Trametinib, 0.4 μM Vemurafenib and 2 nM Trametinib were taken as one basic combination of Vemurafenib and Trametinib (BCVT) in this study. For example, two fold BCVT is the combination of 0.8 μM Vemurafenib and 4 nM Trametinib. Ten fold BCVT is the combination of 4 μM Vemurafenib and 20 nM Trametinib. Then combined GI_50_ (C-GI_50_) of A375, Mel888 and SK–MEL28 cells was assessed by treating these cells with series folds of BCVT. As shown in Fig. 2d, the combined GI_50_ of A375 is about 0.62 BCVT, which means about 0.25 μM Vemurafenib and 1.2 nM Trametinib combination.

To build A375 CIBM resistant cells (A375-P), five fold BCVT (2 μM Vemurafenib and 10 nM Trametinib) was applied. The combined GI_50_ (C-GI_50_) of A375 and A375–P was used to confirm the resistance to CIBM. As shown in Fig. 2e, the combined GI_50_ of A375–P is increased over 30 folds. Hence, A375–P cells showed notable resistance to CIBM. Then A375-P drug resistant cells were used for the further siRNA screening. After transfected with siRNAs as noted in Fig. 2f, the resultant cells were assessed by MTT analysis. It was found that silencing BMK1 showed the most significant ability to inhibit the proliferation of A375-P cells (Fig. 2f). These results are consistent with the previous studies that phospho-BMK1 promotes the proliferation and drug resistance of cancer cells^11^,^13^,^17^. These results also suggested that BMK1 pathway might play an important role in the resistance to CIBM.

### Phosphorylation of BMK1 is associated with the resistance to CIBM

**Figure 3:**
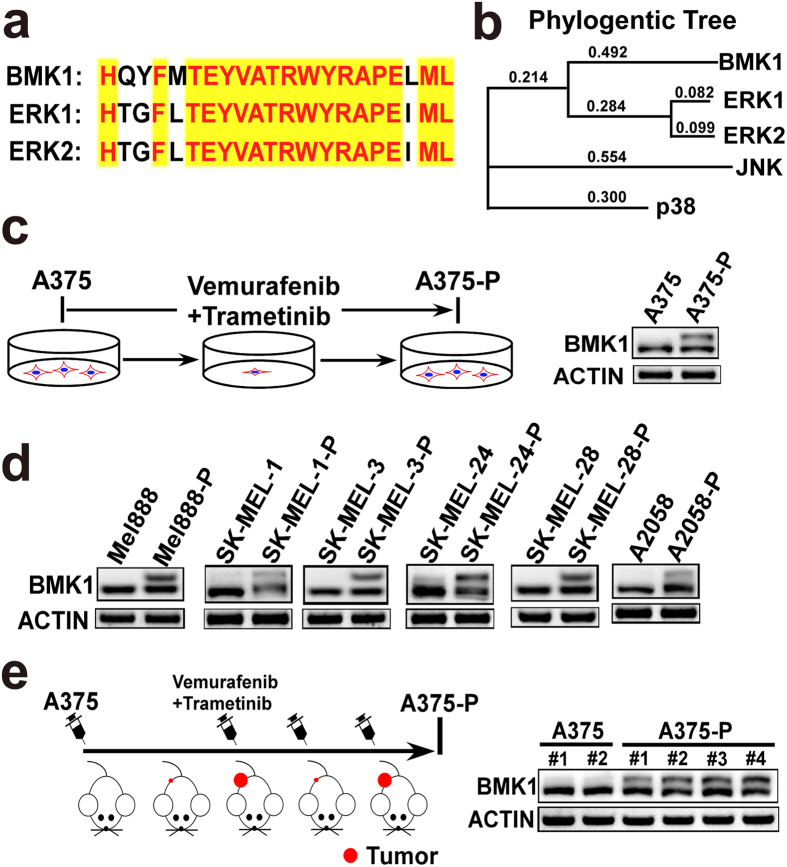
Phosphorylation of BMK1 is associated with the resistance to CIBM. (**a**) The Thr-Glu-Tyr (TEY) activation motif of BMK1 shows significant similarity with ERK1/2. (**b**) Phylogentic tree analysis indicates that BMK1 is the most closely related to ERK1/2. (**c**) Phospho-BMK1 was enhanced by the combined inhibition of BRAF and MEK1/2. Phospho-BMK1 was evaluated in A375-P resistant cells, which were built as described above. (**d**) Several BRAF V699E mutant melanoma cell lines were used to evaluate the phosphorylation of BMK1 in drug resistant cells, which were built as described above. (**e**) The phosphorylation of BMK1 is increased in A375-P xenograft model, compared with A375 xenograft model. Briefly, A375 cells were suspended in DMEM and injected subcutaneously into the flank of Nod/Scid mice. After 2 weeks, the mice were grouped and treated with/without Vemurafenib and Trametinib. The phospho-BMK1 in resulting individual A375 (without Vemurafenib and Trametinib treatment) and A375-P (with Vemurafenib and Trametinib treatment) tumors was evaluated by western blot as noted.

As mentioned above, BMK1 shows the most similar to ERK1/2 (Fig. 3a,b). Meanwhile, BMK1 is well known to promote cell survival and share a bunch of substrates with ERK1/2^11^,^12^. In addition, among the detected upregulated phospho-proteins in this study, BMK1 showed the most significant ability to promote the proliferation of A375-P cells (Fig. 2f). Hence, our study was focused on BMK1.

To evaluate the association between BMK1 and the resistance to CIBM, phosphorylation of BMK1 was first assessed in A375 and A375-P cells. It was found that phospho-BMK1 was increased in A375-P compared with its matched parental control, A375 (Fig. 3c). Moreover, a collection of BRAF V600E mutant melanoma cell lines were used to evaluate the phospho-BMK1 in the CIBM resistant cells, which were built as described above. As shown in Fig. 3d, there was notable upregulation of phospho-BMK1 in 6 of 6 CIBM resistant cell lines (Mel888-P, SK-MEL-1-P, SK-MEL-3-P, SK-MEL-24-P, SK-MEL-28-P and A2058-P). Hence, it was suggested that phosphorylation of BMK1 is associated with the resistance to CIBM. Furthermore, a mouse xenograft model was built to assess the *in vivo* association between BMK1 and the resistance to CIBM. Briefly, A375 cells were injected subcutaneously into the flank of mice. After 2 weeks, the mice were treated with/without CIBM. Consistent with the *in vitro* data, phosphorylation of BMK1 was upregulated in A375-P xenograft tumors as expected (Fig. 3e). Our results suggested that phosphorylation of BMK1 was associated with the resistance to CIBM in melanomas.

### Phosphorylation of BMK1 enhances the resistance to CIBM

**Figure 4:**
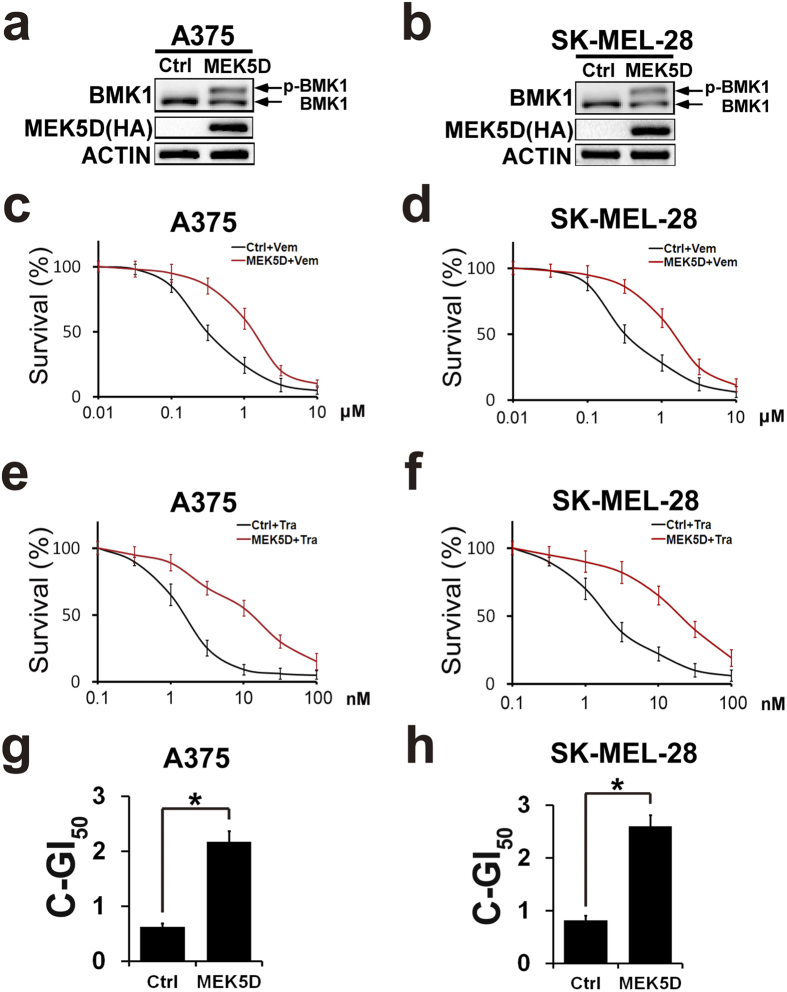
Phosphorylation of BMK1 enhances the resistance to CIBM. (**a**,**b**) A375 and SK-MEL-28 cells were transfected with a constitutively active mutant of MEK5 (HA-MEK5D) and empty vector followed by selection with puromycin. The lysates of stable vector (control) and MEK5D cells were analyzed by western blot using anti-BMK1 and anti-ACTIN antibodies as noted. (**c**,**e**) A375-Ctrl (control) and A375-MEK5D cell growth inhibition curves of Vemurafenib or Trametinib as noted. Briefly, six replicates of A375-Ctrl (control) and A375-MEK5D cells were treated with Vemurafenib or Trametinib for three days at the concentration as noted. Then MTT assays were used to build growth inhibition curves. Unless otherwise stated, three-day MTT assays were used to build growth inhibition curves in this study. (**d**,**f**) SK-MEL-28-Ctrl (control) and SK-MEL-28-MEK5D cell growth inhibition curves of Vemurafenib or Trametinib as noted. (**g**) A375-Ctrl and A375-MEK5D cell combined GI_50_ of Vemurafenib and Trametinib as noted. The combined GI_50_ was assessed as described in Fig. 2d. (**h**) SK-MEL-28-Ctrl and SK-MEL-28-MEK5D cell combined GI_50_ of Vemurafenib and Trametinib.

To investigate the role of phospho-BMK1 in the resistance to CIBM, a constitutively active mutant of MEK5 (MEK5D) was used to phosphorylate and activate BMK1. A375 and SK-MEL-28 cells were transfected with the active mutant (HA-MEK5D) and empty vector followed by selection with puromycin. As showed in Fig. 4a,b, stable expression of MEK5D promoted the phosphorylation of BMK1 in A375 and SK-MEL-28 cells. Then the resultant stable control (empty vector) and MEK5D cells were treated with increasing concentrations of Vemurafenib or Trametinib as noted in Fig. 4c–f. After three days, the number of survival cells was assessed by MTT. Resultant data indicated that MEK5D-phosphorylated BMK1 promoted the resistance to Vemurafenib and Trametinib (Fig. 4c–f). Furthermore, the combined GI_50_ of Vemurafenib and Trametinib was assessed as described above to evaluate the role of BMK1. It was found that phosphorylation of BMK1 increased the combined GI_50_ of CIBM in A375 and SK-MEL-28 cells (Fig. 4g,h).

### Inhibition of BMK1 suppresses the resistance to CIBM

**Figure 5:**
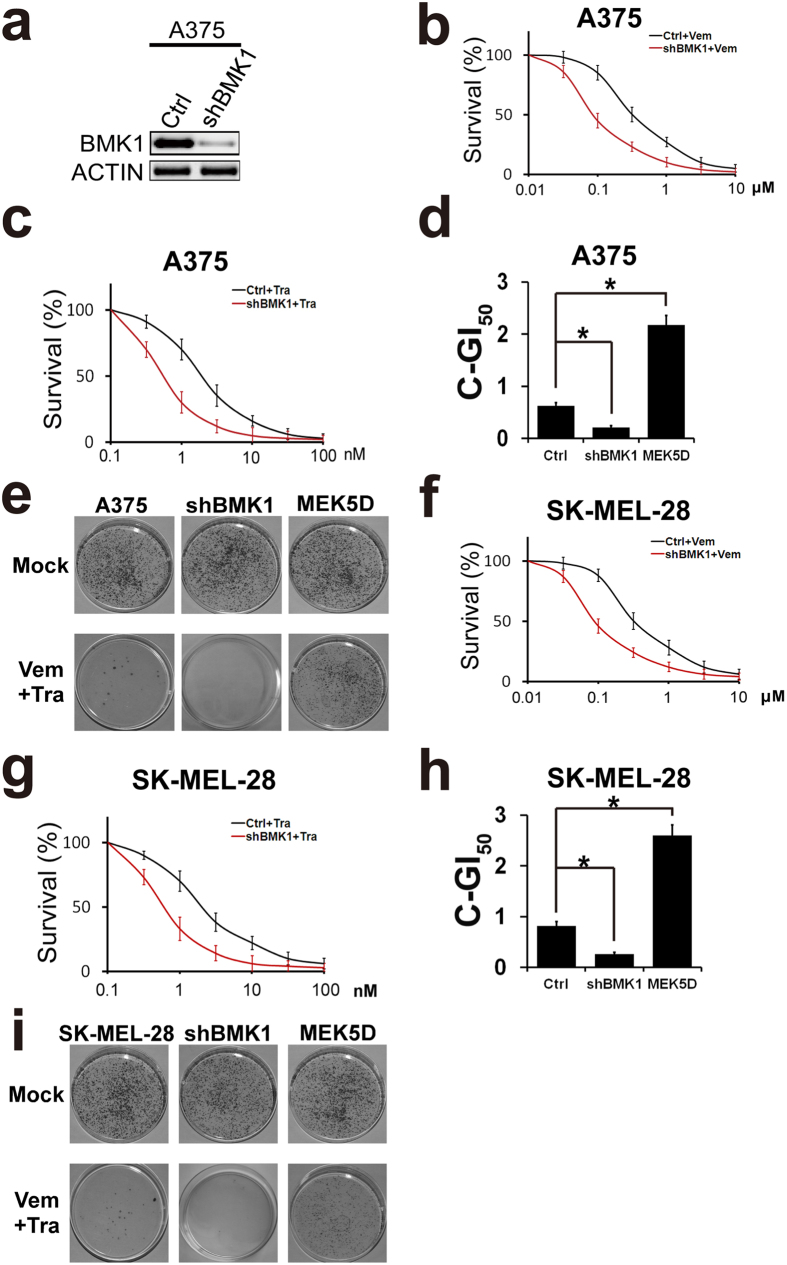
Inhibition of BMK1 by shRNAi suppresses the resistance to CIBM. (**a**) Control and shRNA-mediated knockdown cells were built as described in our previous study^22^. Then the A375 cell lysates were analyzed by western blot using anti-BMK1 and anti-ACTIN antibodies as noted. (**b**,**c**) A375-Ctrl (control) and A375-shBMK1 cell growth inhibition curves of Vemurafenib or Trametinib as noted. (**d**) A375-Ctrl and A375-shBMK1 cell combined GI_50_ of Vemurafenib and Trametinib. (**e**) Colony formation assay of the indicated cell lines. Cells were treated with/without the combination of 2 μM Vemurafenib and 10 nM Trametinib as noted. Resultant cells were stained with crystal violet. (**f**,**g**) SK-MEL-28-Ctrl (control) and SK-MEL-28-shBMK1 cell growth inhibition curves of Vemurafenib or Trametinib as noted. (**h**) SK-MEL-28-Ctrl and SK-MEL-28-shBMK1 cell combined GI_50_ of Vemurafenib and Trametinib.

To further investigate the role of BMK1, BMK1 was knocked down in A375 cells using shRNA as described in previous study (Fig. 5a)^22^. Then resultant control and shBMK1 cells were treated with increasing concentrations of Vemurafenib or Trametinib. Compared with the control cells, shBMK1 cells were more sensitive to BRAF or MEK inhibitor (Fig. 5b,c). Then combined GI_50_ of CIBM was assessed as described above to evaluate the role of BMK1. As shown in Fig. 5d, MEK5D-phosphorylated BMK1 enhanced combined GI_50_, while shRNA-mediated knockdown of BMK1 decreased combined GI_50_. Moreover, colony formation assay was carried out to confirm the role of BMK1 in drug resistance. Consistent with the role of BMK1 in combined GI_50_, MEK5D-phosphorylated BMK1 promoted the drug resistance to CIBM, while shRNA-mediated knockdown of BMK1 decreased the drug resistance (Fig. 5e). Then we expanded these result to SK-MEL-28 cells. Consistent with the results of A375, phospho-activation of BMK1 enhanced drug resistance, while knockdown of BMK1 decreased drug resistance (Fig. 5f–h).

**Figure 6:**
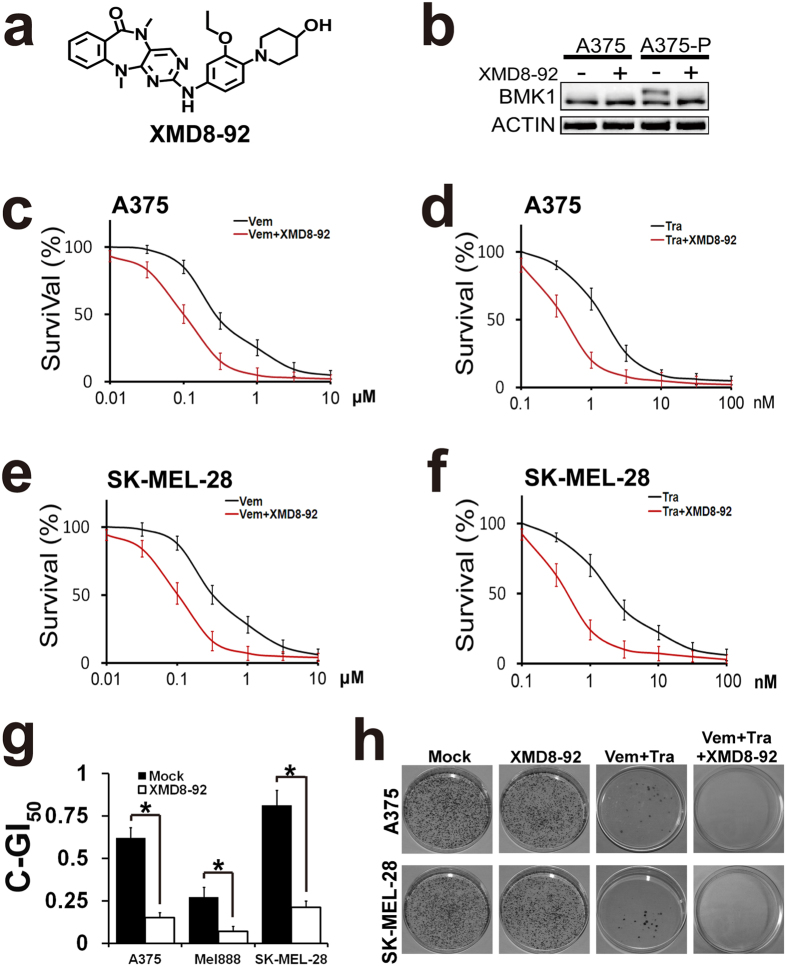
BMK1 inhibitor, XMD8-92, suppresses the resistance to CIBM. (**a**) Chemical structure of BMK1 inhibitor XMD8-92. (**b**) BMK1 inhibitor, XMD8-92 blocks the phosphorylation of BMK1 in A375-P. A375 and A375-P resistant cells were treated with 2 μM XMD8-92 for 1 hr. Then the cell lysates were analyzed by western blot using anti-BMK1 and anti-ACTIN antibodies as noted. (**c**) A375 cell growth inhibition curves of XMD8-92 and/or Vemurafenib as noted. In the presence or absence of 2 μM XMD8-92, A375 cells were treated with Vemurafenib at the concentration as noted. Then three-day MTT assays were used to build growth inhibition curves. (**d**) A375 cell growth inhibition curves of XMD8-92 and/or Trametinib as noted. (**e**) SK-MEL-28 cell growth inhibition curves of XMD8-92 and/or Vemurafenib as noted. (**f**) SK-MEL-28 cell growth inhibition curves of XMD8-92 and/or Trametinib as noted. (**g**) A375, Mel888 and SK-MEL-28 cell combined GI_50_ of Vemurafenib and Trametinib in the presence or absence of 2 μM XMD8-92. (**h**) Colony formation assay of the indicated cell lines. Cells were treated with/without 2 μM Vemurafenib and 10 nM Trametinib in the absence or presence of 2 μM XMD8-92 as noted. Resultant cells were stained with crystal violet.

To confirm the roles of BMK1 inhibition, a small-molecule inhibitor of BMK1, XMD8-92, was applied (Fig. 6a). In our previous study^13^, it has been described that XMD8-92 impairs the tumorigenesis through the inhibition of BMK1 pathway. As shown in Fig. 6b, XMD8-92 blocked the phosphorylation of BMK1 in CIBM resistant cells. To investigate the role of BMK1 in the resistance, growth inhibition curves of Vemurafenib and Trametinib were built in the presence or absence of XMD8-92 as noted. The resultant growth inhibition curves indicated that BMK1 inhibitor synergistically enhanced the ability of Vemurafenib and Trametinib to block the proliferation of A375 (Fig. 6c,d) and SK-MEL-28 (Fig. 6e,f) cells. Then combined GI_50_ of CIBM was assessed as described above to evaluate the role of XMD8-92. As shown in Fig. 6g, XMD8-92 treatment resulted in the decrease of combined GI_50_ of CIBM. In addition, colony formation assay was carried out to evaluate the role of BMK1 in acquirement of drug resistance to CIBM. A375 and SK-MEL-28 cells were treated with/without Vemurafenib and Trametinib in the absence or presence of XMD8-92 as noted in Fig. 6h. Resultant cells were stained with crystal violet (Fig. 6h). It was found that XMD8-92 prolonged the time to acquire the resistance to CIBM (Fig. 6h). Collectively inhibition of BMK1 suppresses the resistance to CIBM.

### Kinome-scale siRNA screening indicated that SRC\MEK5 promotes the phosphorylation of BMK1 in response to CIBM

**Figure 7:**
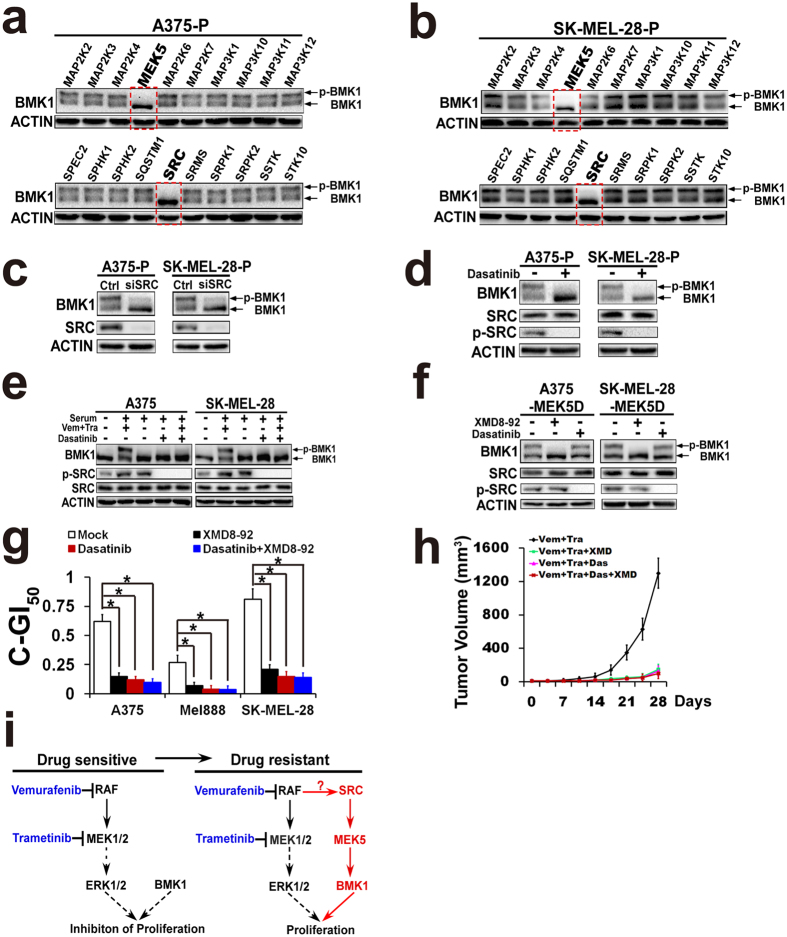
Kinome-scale siRNA screening indicated that SRC\MEK5 promotes the phosphorylation of BMK1 in respons to CIBM. (**a**,**b**) Human siGENOME siRNA library containing 779 kinases (Supplementary Table S4) was used for the screening. After transfected with siRNA for 48 hrs, resultant A375-P and SK-MEL-28-P drug resistant cells were harvested and analyzed by western blotting as noted. (**c**) After transfected with control or siSRC for 48 hrs, resultant A375-P and SK-MEL-28-P drug resistant cells were harvested and analyzed by western blotting as noted. (**d**) A375-P and SK-MEL-28-P cells were treated with/without 2 μM Dasatinib for 1 hr. Resultant cells were harvested and analyzed by western blotting. (**e**) A375 and SK-MEL-28 cells were serum starved overnight followed by treatment with 2 μM Vemurafenib + 10 nM Trametinib, or/and 2 μM Dasatinib for 1 hr as noted. Then cells were stimulated with serum for 1.5 hrs and phosphorylated BMK1 was detected by mobility retardation^27^. Resultant cell lysates were analyzed by western blotting using the antibody as noted. (**f**) A375-MEK5D and SK-MEL-28-MEK5D cells expressing MEK5D were treated with/without 2 μM Dasatinib or 2 μM XMD8-92 for 1 hr. Resultant cells were harvested and analyzed by western blotting as noted. (**g**) A375, Mel888 and SK-MEL-28 cell combined GI_50_ of Vemurafenib and Trametinib in the presence or absence of 2 μM XMD8-92 and/or 2 μM Dasatinib as noted. (**h**) A375 cells were suspended in DMEM and injected subcutaneously into the right flank of 6-week-old Nod/Scid mice. And these tumor-bearing mice were randomized into groups. Mice were injected IP with Vemurafenib (50 mg/kg) (once a day), Trametinib (1 mg/kg) (once a day), XMD8-92 (50 mg/kg) (twice a day), and/or Dasatinib (20 mg/kg) (once a day) as noted. Tumor size was measured twice a week and tumor volume was calculated by using the formula: 0.52 X *L* X *W*^*2*^, where *L* was the longest diameter and *W* was the shortest diameter. (**i**) Scheme for BMK1 enhancing drug resistance to CIBM.

To uncover the mechanisms of activation of BMK1 in response to CIBM, a kinome-scale siRNA screening was first carried out in A375-P and SK-MEL-28-P drug resistant cells. Human siGENOME siRNA library containing 779 kinases (Supplementary Table S4) was used for the screening. After transfected with siRNAs for 48 hrs, resultant A375-P and SK-MEL-28-P cells were harvested and analyzed by western blotting. As shown in Fig. 7a,b, knockdown of MEK5 and SRC showed the most significant ability to inhibit the phosphorylation of BMK1. To confirm the result of siRNA interference of SRC (Fig. 7c), a SRC inhibitor—Dasatinib was applied. Western blotting of resultant cell lysates indicated that Dasatinib indeed blocked the phosphorylation of SRC and BMK1 in A375-P and SK-MEL-28-P drug resistant cells (Fig. 7d). To evaluate the role of SRC\MEK5 in initial response to CIBM, A375 and SK-MEL-28 cells (not drug resistant cells) were treated with/without Dasatinib and/or CIBM for 1 hr as noted in Fig. 7e. Then these cells were treated with/without serum for 1.5 hrs. Western blotting of resultant cell lysates demonstrated that inhibition of SRC also blocked the rapid counteracting phosphorylation of BMK1 in initial response to CIBM (Fig. 7e). To investigate the relation between MEK5 and SRC, A375-MEK5D and SK-MEL-28-MEK5D cells expressing MEK5D (described in Fig. 4a,b) were treated with/without XMD8-92 or Dasatinib as noted for 1 h. Unlike XMD8-92, Dasatinib could not inhibit the phosphorylation of BMK1 in A375-MEK5D and SK-MEL-28-MEK5D (Fig. 7f), which means that SRC is an upstream of MEK5. Consistent with this result, inhibition of SRC by Dasatinib as well as combination with BMK1 inhibitor XMD8-92 sensitized cells to CIBM (Fig. 7g). Further animal data also indicated that XMD8-92 and Dasatinib at least prolonged the time to acquire the resistance to CIBM (Fig. 7h).

To investigate the potential mechanisms of BMK1-mediated resistance to CIBM, we used microarrays (Supplementary Fig. S3a) to identify the genes whose expression was changed after XMD8-29 treatment (Supplementary Table S5). Further enrichment studies of subcellular localization (Supplementary Fig. S3b), molecular function (Supplementary Fig. S3c), biological process (Supplementary Fig. S3d) and pathway analysis (Supplementary Fig. S3e) indicated that inhibition of BMK1 led to hamper the expression of components of MAPK pathways (such as RASGRP1, RASGRP3, IGF1R) and genes associated with cell survival (such as BIRC3). It seems that BMK1 promotes the drug resistance through both potential reactivation of ERK1/2 pathway and enhancement of survival pathways.

## Discussion

BRAF mutant melanomas acquire resistance to anti-BRAF monotherapy via several distinct mechanisms^4^,^5^,^6^, by which ERK1/2 pathway is frequently reactivate. Therefore, it is reasonable to combine inhibition of BRAF and MEK1/2 (CIBM) to bypass these mechanisms. As expected, the CIBM results in a improvement in the clinic, compared with anti-BRAF monotherapy^7^,^8^. But the failure due to drug resistance is still inevitable, which reinforces the importance of understanding the drug resistant mechanisms. In the meantime, it is well known that ERK1/2 pathway regulates numerous cellular processes through phosphorylation. However, little is known about phospho-change of melanomas in response to CIBM. Here, we describe SILAC phosphoproteomic profiling of CIBM, and demonstrate that inhibition of BMK1 might have therapeutic potential for the treatment of melanoma (Fig. 7i).

Our study demonstrated that targeting ERK1/2 pathway induces rapid adaptation of initially drug-responsive melanomas through phospho-regulation of multi pathways. Our phosphoproteomic profiling showed that phosphorylation of 289 sites from 239 proteins was decreased by CIBM, while phosphorylation of 53 sites from 47 proteins was significantly increased. To identify the emergent counteracting pathways, we focused on the upregulated phospho-proteins. siRNA screening demonstrated that the proliferation of CIBM resistant cells was hampered by the knockdown of these upregulated proteins, which are involved in multi survival pathways. Hence, it seems that effective combination therapy to block early counteracting adaptation might be critical.

Furthermore, our study reveals a link between alterations in the MAPK signaling pathways, phospho-activation of BMK1 and resistance to CIBM in melanomas. Our phosphoproteomic data indicated that phosphorylation of 286 proteins was significantly regulated by CIBM. Enrichment analysis of these 286 proteins indicated that both downregulated and upregulated phospho-proteins showed significant enrichment in MAPK pathways (Fig. 1i). Further siRNA screening of upregulated phospho-proteins demonstrated that knockdown of BMK1 or JNK MAPK pathways impaired the drug resistance. In fact, during the preparation of this paper, JNK pathway has been reported as a key mediator of drug resistance in melanoma^23^. Considering the notable ability of BMK1 MAPK pathway to hamper the drug resistance, we focused our study on BMK1.

BMK1, as the most similar kinase to ERK1/2, share a bunch of substrates with ERK1/2^11^,^12^. BMK1 has been reported to regualte multi properties of cancer, including tumorigenesis, chemoresistance^14^, proliferation^15^ and metastasis^16^. Although traditional deletion of BMK1 results in embryonic lethal, conditional knockout of BMK1 in various tissues (such as neurons, hepatocytes, cardiomyocytes, and T and B cells) has no obvious effect on the development, behavior, reproduction and aging of the mouse^24^. In our previous study^13^, we developed a small molecular inhibitor of BMK1, XMD8-92, which is well tolerated and able to suppress the tumorigenesis. In this study, our data suggest that BMK1 is also a target for impairing drug resistance to CIBM. Our data shown here demonstrate that inhibition of BMK1 provides a potential clinically translatable therapeutic strategy for preventing drug adaptive resistance mediated through BMK1.

In addition, our study reinforces the roles of tyrosine kinases in drug resistance. Our data indicated that the upregulated phosphosites was significantly enriched in phospho-tyrosine sites (*p* value 0.0049), which is consistent with the previous studies—tyrosine kinase-activated survival pathways promote the drug resistance^19^,^20^,^21^. The enrichment of phospho-tyrosine sites suggests potential roles of tyrosine kinases in drug resistance. Consistent with the enrichment result of tyrosine sites, kinome scale siRNA screening indicated that SRC\MEK5 cascade promotes the phosphorylation of BMK1 in response to CIBM in melanoma. It seems that inhibition of SRC and/or BMK1 provides a potential therapeutic strategy (Fig. 7i).

However, some limitations of this study should be noted. Firstly, we only concentrate on BMK1 in this study, although the phosphorylation of multi proteins is upregulated in response to CIBM. As shown in Fig. 2f, knockdown of c-JUN also significantly inhibited the proliferation of the resistant cells. During the preparation of this paper, JUN has been reported as a key mediator of drug resistance to BRAF inhibitor^23^, which is consistent with our study. Secondly, due to the complexity of the drug resistant mechanisms, it is definite that some proteins involving in the drug resistance are not detected in this study. Despite these limitations, this study can clearly indicate that targeting ERK1/2 pathway induces rapid adaptation of initially drug-responsive melanomas through phospho-regulation of multi pathways, and inhibition of BMK1 has therapeutic potential for the treatment of melanoma.

Collectively, we not only provide a global phosphoproteomic profiling of CIBM in melanoma, but also demonstrate that BMK1 contributes to the drug resistance and might serve as a potential drug target.

## Methods

### Cell culture and transfection

A375, A2058, Mel888, SK-MEL-1, SK-MEL-3, SK-MEL-24 and SK-MEL-28 melanoma cell lines were from the ATCC. All of these cells contain the BRAF (V600E) mutant. A375, Mel888, A2058 HeLa and A549 cells were maintained in Dulbecco’s modified Eagle’s medium (DMEM) containing 10% FBS, 2 mM glutamine, 100 U/mL penicillin and streptomycin at 37 °C under a humidified atmosphere of 5% CO_2_. SK-MEL-1, SK-MEL-24 and SK-MEL-28 cells were maintained in Eagle’s Minimum Essential Medium containing 10% FBS, 2 mM glutamine, 100 U/mL penicillin and streptomycin. SK-MEL-3 were maintained in McCoy’s 5a Modified Medium containing 10% FBS, 2 mM glutamine, 100 U/mL penicillin and streptomycin. Transfections were performed using lipofectamine 2000 (Thermo Fisher Scientific Inc., Waltham, MA USA) according to the manufacturer’s instruction.

### SILAC-labeling and mass spectrometry analysis

SILAC-labeling and mass spectrometry analysis were performed as described in our previous study^13^,^18^,^25^. Briefly, GIBCO SILAC DMEM basal cell culture medium (Invitrogen, Carlsbad, CA, USA) containing 2 mM L-glutamine, 10% dialyzed fetal bovine serum (FBS) (Invitrogen), 100 U/mL penicillin and streptomycin was supplemented with 100 mg/L L-lysine and 20 mg/L L-arginine or 100 mg/L [U-^13^C_6_]-L-lysine and 20 mg/L [U-^13^C_6_, ^15^N_4_]-L-arginine (Invitrogen) to make the “light” or “heavy” SILAC media, respectively. A375 cells were obtained from American Type Culture Collection (ATCC) and cultured in SILAC medium for over 16 generations to ensure almost 100% incorporation of labeled amino acids. Light cells were pretreated with 2 μM Vemurafenib and 10 nM Trametinib for 1 hr. After treated with serum for 1.5 hrs, heavy and light cell lysates were combined in a 1:1 ratio and analyzed by mass spectrometer as described in previous study^18^.

### Immunoblotting

Immunoblotting was carried out as described in our previous study^13^,^18^,^22^. Briefly, cells were harvested in RIPA buffer. Proteins from total cell lysates were resolved by SDS–polyacrylamide gel electrophoresis, then transferred to nitrocellulose membrane, blocked in 5% nonfat milk and blotted with the appropriate antibody as noted.

### Antibodies and plasmids

Anti-HA (Cat. number: 3724), anti-BMK1 (Cat. number: 3372), anti-ERK1/2 (Cat. number: 9102), anti-phosho-ERK1/2 (Thr202/Tyr204) (Cat. number: 4376), anti-phosho-Jun (Ser63) (Cat. number: 2361), anti-Jun (Cat. number: 2315), anti-SRC (Cat. number: 2109), anti-phosho-SRC (Tyr416) (Cat. number: 2101) and anti-ACTIN (Cat. number: 4967) antibodies were from Cell Signaling (Cell Signaling, Beverly, MA USA). pCDNA3 HA-tagged MEK5D vectors was described in our previous study^13^. pGIPZ-BMK1 encoding shRNA against human BMK1, and pGIPZ-nontarget shRNAs were from Open Biosystems as described in the previous study^26^.

### siRNA and shRNA analysis

siRNA and shRNA analysis were carried out as described in our previous study^13^,^18^,^26^. Human siGENOME siRNA Library (SMARTpool) from Dharmacon (Dharmacon, Lafayette, CO, USA) containing 779 kinases (Supplementary Table S4) was used for the siRNA screening.

### Colony formation assay

For clonogenic assays, 5 × 10^3^ cells were plated in 10 cm dish in fresh media. After 24 h, cells were treated with inhibitors as noted. Then cells were stained with 0.5% (w/v) crystal violet in 70% ethanol.

### Xenografts

The following animal-handling procedures were approved by the Animal Care and Use Committee of DaLian Medical University. All methods were performed in accordance with the relevant guidelines and regulations of the Animal Care and Use Committee of DaLian Medical University. Xenograft models were carried out as previously described^13^. Briefly, 1 × 10^7^ cultured A375 cells were suspended in DMEM and injected subcutaneously into the right flank of 6-week-old Nod/Scid mice. Resultant mice were injected IP with drug as noted. Tumor size was measured twice a week by caliper and tumor volume was calculated by using the formula: 0.52 X *L* X *W*^*2*^, where *L* was the longest diameter and *W* was the shortest diameter.

### Statistical analysis

*p* values were calculated using the Student’s *t*-test or Chi-square test as noted.

## Additional Information

**How to cite this article:** Song, C. *et al*. Targeting BMK1 Impairs the Drug Resistance to Combined Inhibition of BRAF and MEK1/2 in Melanoma. *Sci. Rep.* **7**, 46244; doi: 10.1038/srep46244 (2017).

**Publisher's note:** Springer Nature remains neutral with regard to jurisdictional claims in published maps and institutional affiliations.

## Supplementary Material

Supplementary Information

Supplementary Dataset 1
