# Corrigendum: Targeting BMK1 Impairs the Drug Resistance to Combined Inhibition of BRAF and MEK1/2 in Melanoma

**DOI:** 10.1038/srep46833

**Published:** 2017-05-26

**Authors:** Chengli Song, Lina Wang, Qiang Xu, Kai Wang, Dan Xie, Zhe Yu, Kui Jiang, Lujian Liao, John R. Yates, Jiing-Dwan Lee, Qingkai Yang

Scientific Reports 7: Article number: 4624410.1038/srep46244; published online: 04 07 2017; updated: 05 26 2017

The Acknowledgements section in this Article is incomplete.

“The authors thank Bingwen Lu for technical assistance with phosphoproteomic analysis. This study was supported by grants from the National Natural Science Foundation of China (NSFC Nos 31471328 and 81622040 to Qingkai Yang). This study was also supported by grants from the National Natural Science Foundation of China (NSFC Nos 81502622 to Lina Wang)”.

should read:

“The authors thank Bingwen Lu for technical assistance with phosphoproteomic analysis. This study was supported by grants from the National Natural Science Foundation of China (NSFC Nos. 31471328 and 81622040 to Qingkai Yang). This study was supported by a grant from the National Natural Science Foundation of China (NSFC Nos. 81502622 to Lina Wang). This study was also supported by a grant from the National Institutes of Health (P41 GM103533 to John Yates)”.

