# Supplementary material for: Targeting BMK1 Impairs the Drug Resistance to Combined Inhibition of BRAF and MEK1/2 in Melanoma
Source: Sci Rep. 2017 Apr 7;7:46244. doi: 10.1038/srep46244 (PMC5384194; doi:10.1038/srep46244)
Supplement: Supplementary Information [file srep46244-s1.pdf]

Targeting BMK1 Impairs the Drug Resistance to Combined Inhibition of BRAF and MEK1/2 in Melanoma

Chengli Song[1], Lina Wang[1], Qiang Xu[1], Kai Wang[1], Dan Xie[1], Zhe Yu[1], Kui Jiang[1], Lujian Liao[2], John R. Yates III[3], Jiing-Dwan Lee[4] & Qingkai Yang[1]

Supplementary Figure legends

Supplementary Figure S1. Phosphoproteomic profiling revealed that the upregulated phosphosites were enriched in phospho-tyrosine sites and MAPK pathways. (**a**) Number of gene enriched according to subcellular localizations as noted. (**b**) Percentage of gene enriched according to subcellular localizations as noted. (**c**) Number of gene enriched according to molecular functions. (**d**) Percentage of gene enriched according to molecular functions. (**e**) Number of gene enriched according to biological processes. (**f**) Percentage of gene enriched according to biological processes.

Supplementary Figure S2. Phosphorylation of ERK1/2 in CIBM resistant cell lines in Fig. 3c and 3d. Phospho- ERK1/2 was evaluated using western blotting as noted.

Supplementary Figure S3. Microarray analysis of A375-P cells treated with/without BMK1 inhibitor, XMD8-92. (**a**) Heatmap of microarray assay data from three individual experiments. A375-P cells were treated with/without 2 μM XMD8-92 for 24 hrs. RNA was extracted using the miRNeasy Mini Kit from QIAGEN (QIAGEN, Hilden, Germany) according to the manufacturer's instruction. Microarray experiments were performed in triplicate as noted. And the microarray data are described in Supplementary Table S5. (**b**) Number of gene enriched according to subcellular localization as noted. (**c**) Number of gene enriched according to molecular function. (**d**) Number of gene enriched according to biological process. (**e**) Number of gene enriched according to pathway analysis.

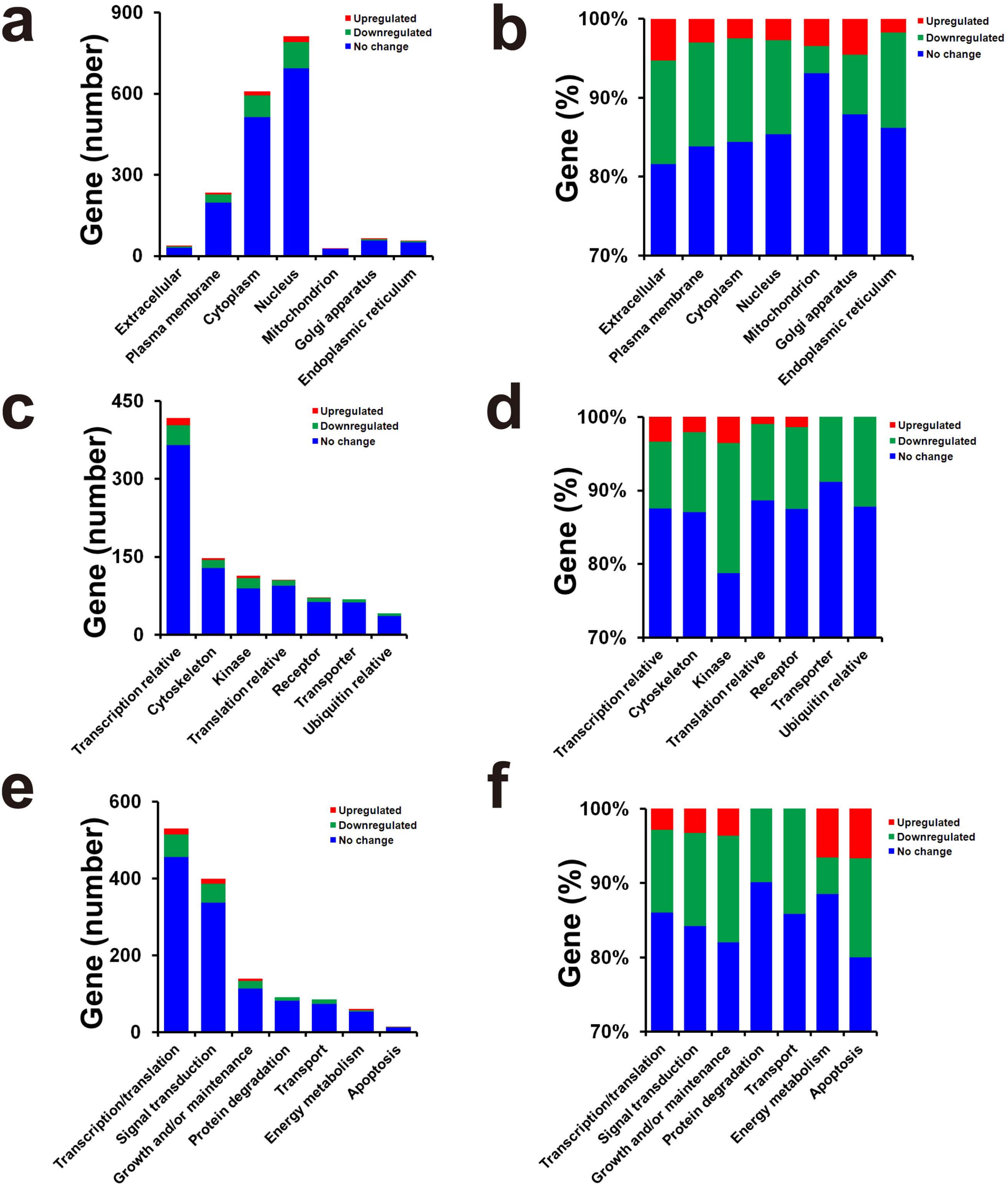

a
Gene (number)
900
600
300
0
Upregulated
Downregulated
No change
Extracellular
Plasma membrane
Cytoplasm
Nucleus
Mitochondrion
Golgi apparatus
Endoplasmic reticulum
b
Gene (%)
100%
90%
80%
70%
Upregulated
Downregulated
No change
Extracellular
Plasma membrane
Cytoplasm
Nucleus
Mitochondrion
Golgi apparatus
Endoplasmic reticulum
c
Gene (number)
450
300
150
0
Upregulated
Downregulated
No change
Transcription relative
Cytoskeleton
Kinase
Translation relative
Receptor
Transporter
Ubiquitin relative
d
Gene (%)
100%
90%
80%
70%
Upregulated
Downregulated
No change
Transcription relative
Cytoskeleton
Kinase
Translation relative
Receptor
Transporter
Ubiquitin relative
e
Gene (number)
600
400
200
0
Upregulated
Downregulated
No change
Transcription/translation
Signal transduction
Growth and/or maintenance
Protein degradation
Transport
Energy metabolism
Apoptosis
f
Gene (%)
100%
90%
80%
70%
Upregulated
Downregulated
No change
Transcription/translation
Signal transduction
Growth and/or maintenance
Protein degradation
Transport
Energy metabolism
Apoptosis

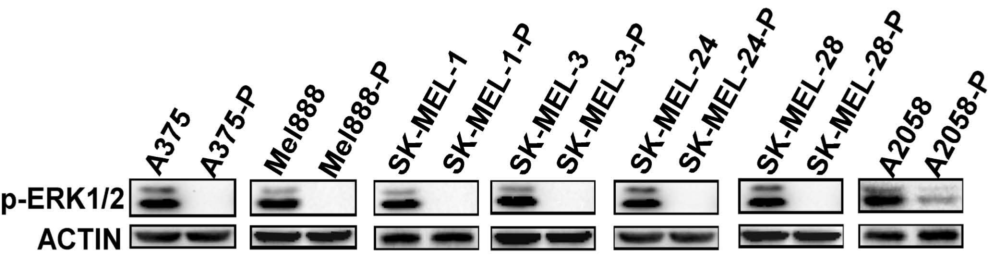
A375
A375-P
Mel888
Mel888-P
SK-MEL-1
SK-MEL-1-P
SK-MEL-3
SK-MEL-3-P
SK-MEL-24
SK-MEL-24-P
SK-MEL-28
SK-MEL-28-P
A2058
A2058-P
p-ERK1/2
ACTIN

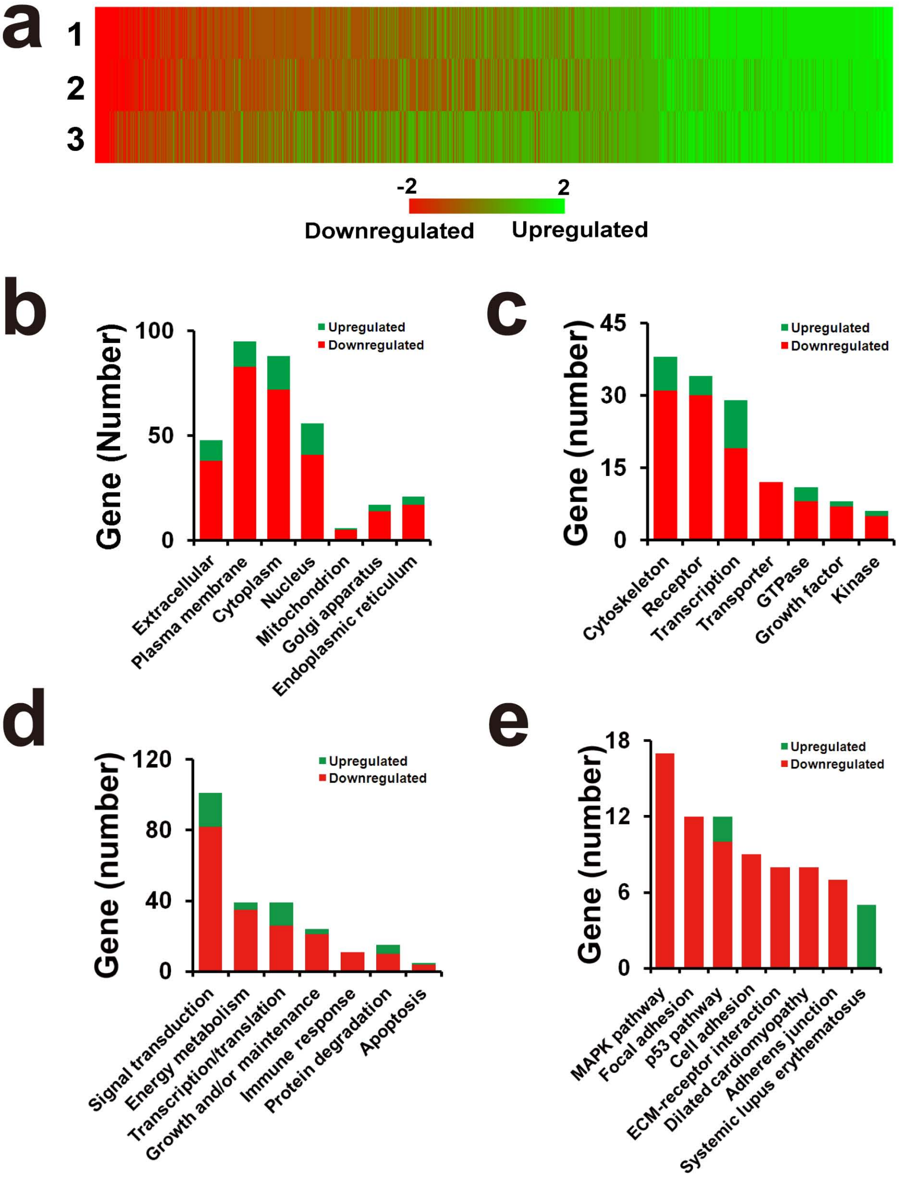
a
1
2
3
-2
2
Downregulated
Upregulated
b
Gene (Number)
100
50
0
Upregulated
Downregulated
Extracellular
Plasma membrane
Cytoplasm
Nucleus
Mitochondrion
Golgi apparatus
Endoplasmic reticulum
c
Gene (number)
45
30
15
0
Upregulated
Downregulated
Cytoskeleton
Receptor
Transcription
Transporter
GTPase
Growth factor
Kinase
d
Gene (number)
120
80
40
0
Upregulated
Downregulated
Signal transduction
Energy metabolism
Transcription/translation
Growth and/or maintenance
Immune response
Protein degradation
Apoptosis
e
Gene (number)
18
12
6
0
Upregulated
Downregulated
MAPK pathway
Focal adhesion
p53 pathway
Cell adhesion
ECM-receptor interaction
Dilated cardiomyopathy
Adherens junction
Systemic lupus erythematosus
